# Are we truly helping those in need: A comprehensive assessment of semen technologies in wild bird species

**DOI:** 10.1007/s10531-026-03397-7

**Published:** 2026-06-29

**Authors:** Marcel Henrique Blank, Julia Roismann, Matheus Moraes Azevedo, Ricardo Jose Garcia Pereira

**Affiliations:** 1 The Roslin Institute and Royal (Dick) School of Veterinary Studies, University of Edinburgh, Roslin, UK; 2 Department of Animal Reproduction, School of Veterinary Medicine and Animal Science, University of São Paulo, Pirassununga, Brazil

**Keywords:** Conservation, Aves, Reproduction, Captive breeding, Assisted reproductive technologies

## Abstract

Semen technologies (STs) have been increasingly used in birds as a tool to overcome difficulties commonly faced by conservation breeding programs. Yet their application faces challenges not only related to avian physiology but also in terms of efficiency and welfare of the animals being handled. Significant advances have been made in some taxonomic groups in recent decades. Nevertheless, these approaches are not as widely disseminated as they are in mammals. Here, we compiled data from 178 scientific articles on STs to better understand the distribution of research among taxonomic groups and reproductive areas, the reasons for this arrangement, and discuss the future priorities for making these tools more effective within global bird conservation. Most available information refers exclusively to semen collection (42%, i.e., methods, parameters, etc.), while a small fraction of the literature describes the use of the complete cycle of STs (16%). These figures highlight that, despite decades of research, refinement in avian STs remains unbalanced with more emphasis on acquiring basic knowledge about semen/sperm than on integrative application of biotechnologies. We observed that STs have been applied to less than 2% of bird species, mostly targeting non-threatened species often chosen for their charisma, monetary value, or ease of maintenance in captivity. This survey provides insights for researchers, breeders, zoos, organizations and funding agencies worldwide to reflect on future paths and guidelines for the development of reproductive technologies in birds making them more useful in preserving species truly in need.

**Supplementary Information:**

The online version contains supplementary material available at 10.1007/s10531-026-03397-7.

## Introduction

Human activities have played a pivotal role in the global decline of biodiversity, with species loss occurring at a rate tens to hundreds of times faster than the average of the last 10 million years (Lees et al. 2025). Conservation initiatives came too late to prevent the extinction of some birds such as the cryptic treehunter (*Cichlocolaptes mazarbarnetti*) and the Alagoas foliage-gleaner (*Philydor novaesi*), but breeding programmes have given hope to other species. For instance, recent attempts to reintroduce the Alagoas curassow (*Pauxi mitu*) and the Spix’s macaw (*Cyanopsitta spixii*) into the wild confirm the success of captive breeding even in species from developing countries with severe genetic bottlenecks (Francisco et al. 2021; Purchase et al. 2024). Efforts to tackle this biodiversity crisis must be as prompt, integrated and effective as possible, and although reproductive technologies are not often considered environmental protection measures, they have the potential to ensure the genetic variability of populations through germplasm banks. A well-known example of this contribution is the recovery of the peregrine falcon in the mid-1960s, when techniques such as artificial insemination and incubation enabled the release of approximately 6,000 birds over 20 years (Enderson et al. 1998).

Within this framework, there are several circumstances in bird conservation programs that may require the use of semen-related technologies (STs) including the inability of a pair to mate naturally (due to physical or behavioral issues), maximizing fertility, producing more offspring from a given individual, distances between key individuals or populations, creating genetic backups, etc. Such applicability has motivated the employment of STs like artificial insemination and semen cryopreservation in more than 40 nondomestic species over the years (e.g., waterfowl, raptors, cranes, passerines, parrots and ratites) (Barna et al. 2020). Nevertheless, various obstacles, ranging from high contamination and small volumes of seminal samples to the inefficiency of avian sperm freezing protocols, have greatly limited the broader application of STs in wild birds. Improvements in these approaches, in turn, depend on time as well as human and financial capital but research in zoos in the reproductive field has historically been biased toward mammals. A similar scenario was recently described by Kaplan (2022), who state that welfare studies in zoos and aquariums in the past decade accounted for less than 10% despite the class representing 30% or more of species on display. Furthermore, existing research on birds seems to focus on larger species with great public appeal, often leading to the captive breeding of birds without conservation purposes.

In view of the above, an overall analysis covering seven decades of work with STs (i.e., semen collection and evaluation, fresh semen storage, semen cryopreservation, and artificial insemination) was conducted to identify trends in terms of study topics, taxonomic groups, bird sizes, diet, threat levels, among others. The paper discusses not only which taxonomic groups have benefited the most and the least from the current body of knowledge, but also identifies important gaps in STs research, evidencing marked disparities in the representation of bird orders and families in the literature. More specifically, this review sought to identify which taxonomic groups have effectively benefited from the application of these biotechnologies and to explore why some groups have been more represented than others in the literature. We believe that these considerations can change decision-making regarding research, support, and funding strategies for a more fruitful partnership among academic institutions, government, private organization and zoos towards the preservation of threatened species.

## Materials and methods

### Dataset screening

A dataset on semen technologies (STs) was created based solely on peer-reviewed articles dealing with semen collection and analysis, liquid semen storage, semen cryopreservation, and artificial insemination in wild birds (i.e., any species excluding domestic birds such as chickens, turkeys, quails, ducks, and geese). This information was achieved by performing searches in four online databases of peer-reviewed publications (Web of Science, Google Scholar, JSTOR and PubMed) using the names of bird genera, families and orders as well as general terms such as “Bird”, “Avian” or specific terms like raptors, birds of prey, parrots, waterfowl, songbirds, among others, in combination with the following keywords: semen, ejaculate, spermatozoa, collection, massage, electrostimulation, cooperative, artificial insemination, cryopreservation and freezing. All articles were also screened according to subject matter, excluding any that did not provide at least one relevant piece of information regarding semen characteristics (i.e., sperm volume, concentration and motility). This measure was taken in order to avoid an unbalanced analysis given the enormous number of articles addressing sperm collection in Passeriformes without any relation to the application of assisted reproductive technologies (e.g., studies of sperm competition in birds such as that by Lüpold et al. 2009).

## Taxonomy and Extinction Risk

The species addressed in the manuscripts were taxonomically classified at the order level according to the Birds of the World platform, Cornell Lab of Ornithology (https://birdsoftheworld.org*)*, and subsequently clustered into higher clades based on taxonomic reassignment published by Stiller et al. (2024). The extinction risk of each species was established following the classification provided by the International Union for Conservation of Nature (IUCN; https://www.iucnredlist.org*)* and divided into seven threat categories: data deficient (DD), least concern (LC), near threatened (NT), vulnerable (VU), endangered (EN), critically endangered (CR), and extinct in the wild (EW).

## Diet and Body Weight

The diet classification followed the compilation made by Wilman et al. (2014) with modifications. These authors proposed a semi-quantitative approach based on the relative importance of the consumed items, with the final classification representing the main diet components. However, species of the families Cracidae, Ramphastidae, and Psittacidae initially classified by Wilman et al. (2014) as Fruit/Nectar feeders, we classified them as Fruit feeders since they were rarely observed consuming nectar. Similarly, species classified by these authors as Vertebrate/Fish/Scavenger feeders (i.e., vertebrates, fishes, and carrion) were considered as Vert feeders only. As information on the diet of the *Chlamydotis macqueenii* were not available, we classified it in the same way as the closest species (*Chlamydotis undulata* - omnivorous). The body weights of each species were calculated as the average between values described for males and females in the Birds of the World platform of the Cornell Lab of Ornithology (https://birdsoftheworld.org*).*

## Modeling Procedures and Statistical Analysis

In order to verify whether the application of STs is biased due to taxonomy, extinction risk, body weight or diet, dietary data (fruit, invertebrate, nectar, omnivore, seed and vertebrate) were treated as categorical explanatory variables, while data on body weight and extinction risk were treated as continuous explanatory variables. Body weight data were log-transformed for statistical analysis. In the case of the extinction risk of a family we used a threat scale (Trel) which was calculated using the formula: Trel = Ʃ (n*Tval^LC^+n*Tval^NT^…+n*Tval^EW^)/s, where *n* is the total number of species in that threat level, *Tval* is the value assigned to the threat level, and *s* is the total number of species in the family. For the *Tval* determination, values between 0 and 1 were assigned to each threat category (least concern [LC], near threatened [NT], vulnerable [VU], endangered [EN], critically endangered [CR], and extinct in the wild [EW]) using the logarithmic trendline. We decided to use logarithmic scale instead of the linear one because in hypothetical scenarios the latter yielded fewer representative scores. For example, on the linear scale with weights of 1 for LC, 0.8 for NT, 0.6 for VU, 0.4 for EN, 0.2 for CR, and 0.0 for EW, a family composed entirely of CR species would receive a final score of 0.2, which we considered insufficient to reflect the severity of this condition. Therefore, we adopted a logarithmic scale to ensure that each successive category reflected a significant worsening of the conservation status. Thus, *Tval* was calculated according to the equation “*Y = −0.551 ln(X) + 1.011; R² = 0.9991*”, where *Y* corresponds to *Tval* and *X* represents each threat category level (LC = 1, NT = 2, VU = 3, EN = 4, CR = 5, and EW = 6). These measures generated the following *Tval* values: 1 for LC, 0.63 for NT, 0.41 for VU, 0.25 for EN, 0.12 for CR and 0.02 for EW.

Species classified as DD (data deficient) were included in the total number of species. This approach allowed for the standardization of conservation status for each family regardless of the number of species per threat level, where the highest values on the scale (close to 1) indicate the most threatened families.

The study scale (i.e., the achievements obtained by the STs) in threatened species was calculated by the formula: Srel = Ʃ(_VU_catscore*F+_EN_catscore*F+_CR_catscore*F+_EW_catscore*F)/T, where *catscore* is the value referent to number of species studied in the threat category (i.e., VU, EN, CR, EW) divided by total number of species in that threat category and multiplied for total of species listed in all threat categories. Then, this value was multiplied by *F* that is a correction factor calculated to assign a weight according to the different threat categories. In this correction factor, scores were assigned to each threat category: 1 for vulnerable, 2 for endangered, 3 for critically endangered and 4 for extinct in the wild. Then, F-values were calculated dividing the threat level score by the sum of the scores (≤ 10) of all threat levels observed in that family. In the end, the summation notation was divided by T that represents the total number of species threatened in the family to find the study scale value (Srel). In this way, it was possible to access the true contribution of STs to endangered species by family, where the highest values (close to 1) on this study scale demonstrate that a greater number of endangered species in that family benefited from advances in STs. Trel and Srel values were not calculated for families Ramphastidae, Rheidae, and Casuariidae because none of their species fall into any of the threat levels. Details of the methodology, including the rationale and formula used to develop the threat scale and study scale for each family, can be found in the Supplementary material (Excel file, “Dataset Heatmap” sheet). One-way ANOVA followed by Tukey’s multiple comparisons test was performed between log-transformed body weight and STs techniques (i.e., semen collection (SC), artificial insemination (AI), and sperm cryopreservation (CR)) using GraphPad Prism version 10.0 for Mac (GraphPad Software, Boston, Massachusetts, USA).

## Results

**Fig. 1:**
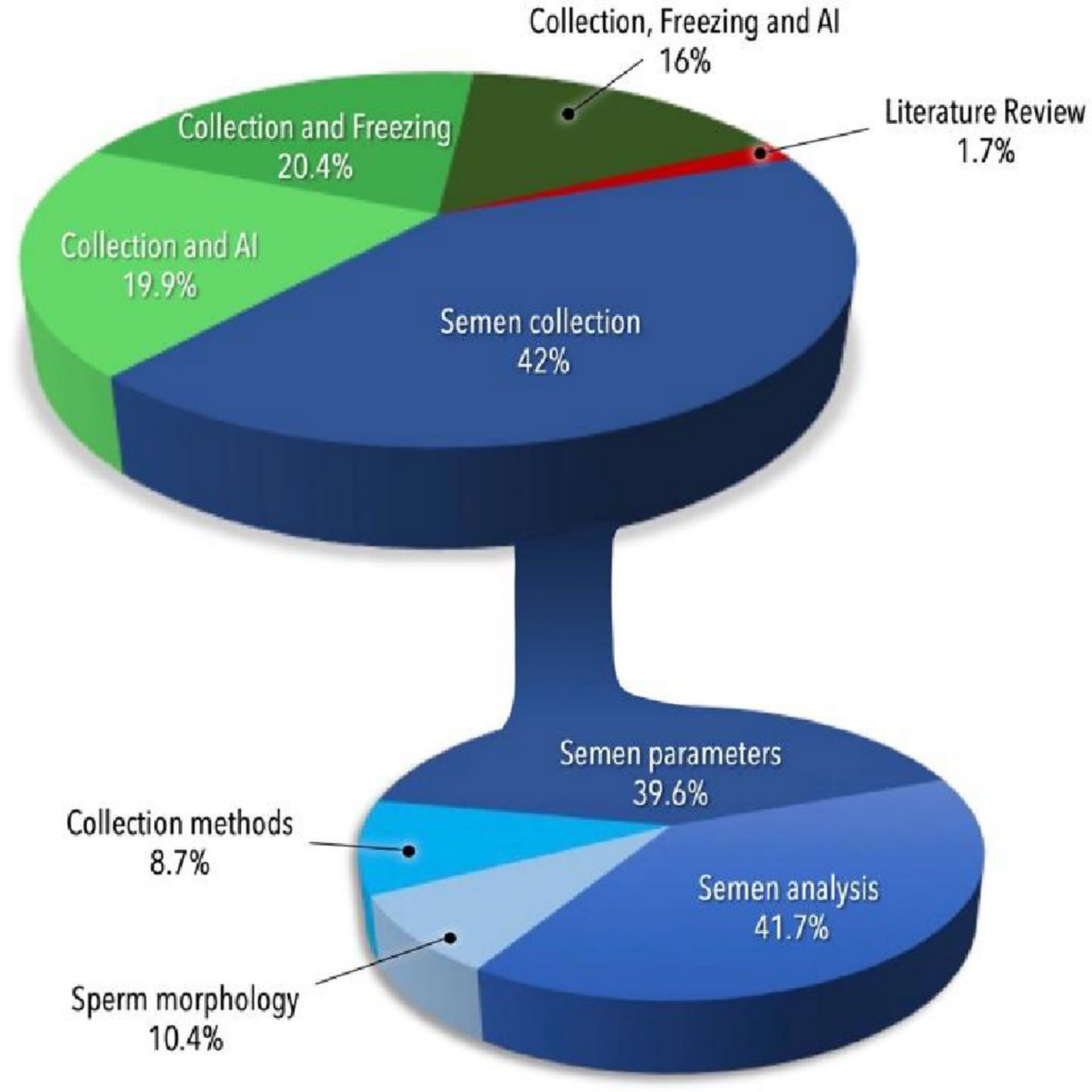
Distribution of the studies involving semen technologies (STs) in wild birds conducted between 1960 and 2024 according to their scientific purposes. The upper graph divides the studies between semen collection only, semen collection associated with artificial insemination (with fresh or cooled semen), semen collection followed by cryopreservation, semen collection with cryopreservation and subsequent artificial insemination, and literature reviews. The lower graph details the objectives of studies dealing only with semen collection, according to the following criteria: (a) *Semen parameters* refers to studies with only macroscopic data and, sometimes, sperm concentration; (b) *Semen analysis* refers to studies with more complete data, such as sperm movement characteristics, viability, defects, among others; (c) *Collection methods* refers to studies that only describe or compare techniques for obtaining ejaculates and their respective efficiencies in avian species; and (d) Sperm morphology refers to studies that only perform microscopic characterization of sperm cells in one or more bird species

Our survey found the production of 178 peer-reviewed articles involving STs in wild birds over the last seven decades, resulting in 403 data points from 222 bird species among 33 families. As expected, the first four decades were marked by a scarcity of literature on the subject (less than 8 studies per decade), but from the 1990 s onwards there has been a growing number of studies, reaching a maximum of 72 articles from 2011 to 2020 (although from 2021 to date, production appears to be following the same trend as the previous decade). A more in-depth analysis of the purposes of the ST studies revealed that most of them only focused on semen collection (42%) aiming to test different collection methods, determine macroscopic semen characteristics, investigate sperm morphology or gather more detailed seminal parameters (Fig. 1). Unfortunately, lower percentages were found regarding the application of semen collection in conjunction with other STs (e.g. artificial insemination and sperm freezing, 19% and 20%, respectively), and even lower the ratio of studies combining the use of these three technologies (16%). In addition, the data showed that the most widely used collection method in the reviewed literature was abdominal massage (65.9%), followed by cooperative collection (i.e., ejaculation of imprinted males into devices such as mannequins, hats, etc., 14.1%), electroejaculation (13.6%), and other alternative techniques (e.g., urodeum stimulation, semen recovered from the testes or vas deferens of necropsied males, etc., 1.9%). Some publications did not describe how the semen was collected (4.5%).

**Fig. 2:**
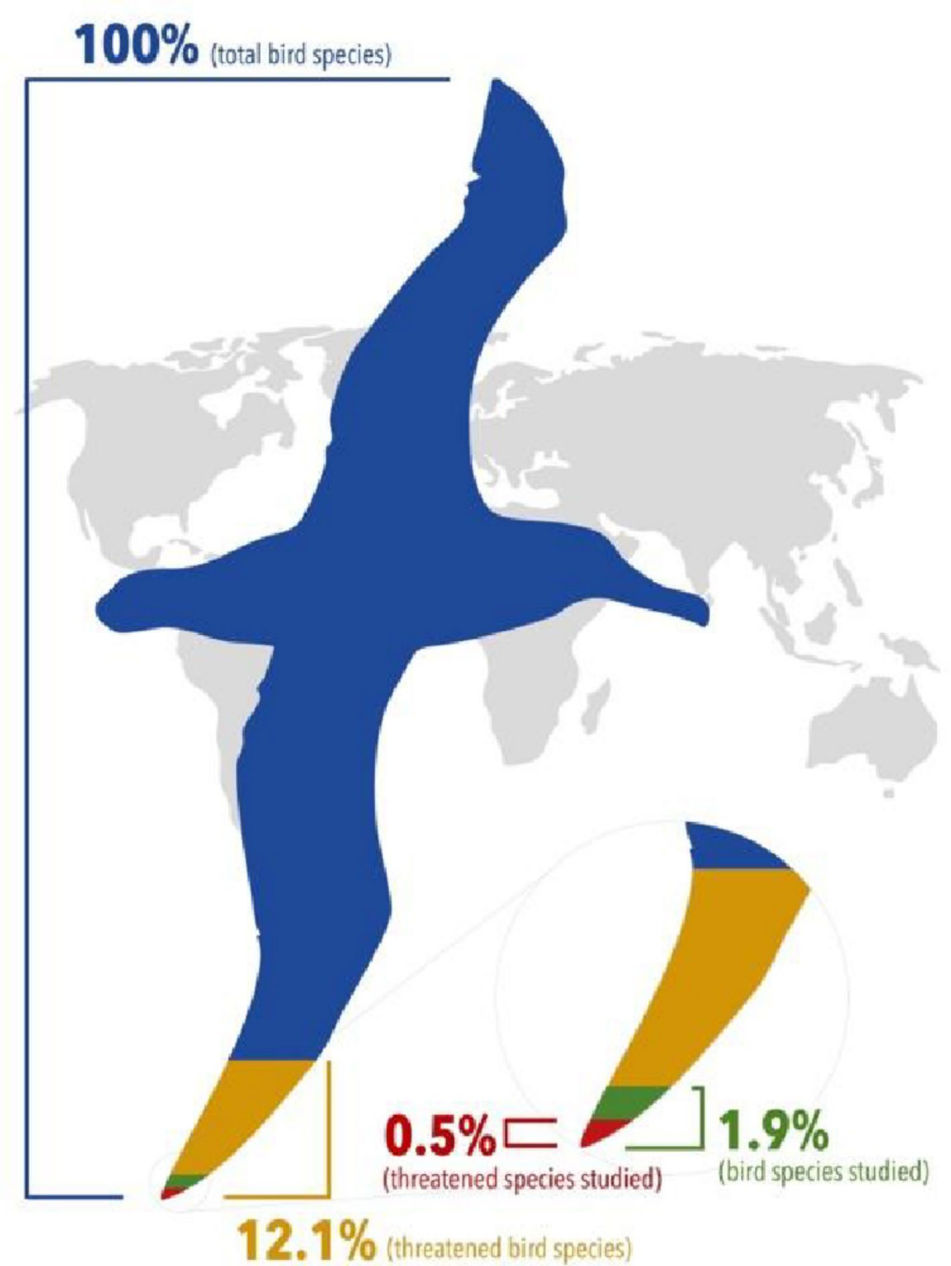
Graphical representation of the percentages of species and threatened species studied for any semen technologies (STs) in relation to the total number of bird species in the world (symbolized by the albatross). The magnification (circle) details the percentages of species addressed by the STs. Data sourced from IUCN Red List

**Fig. 3:**
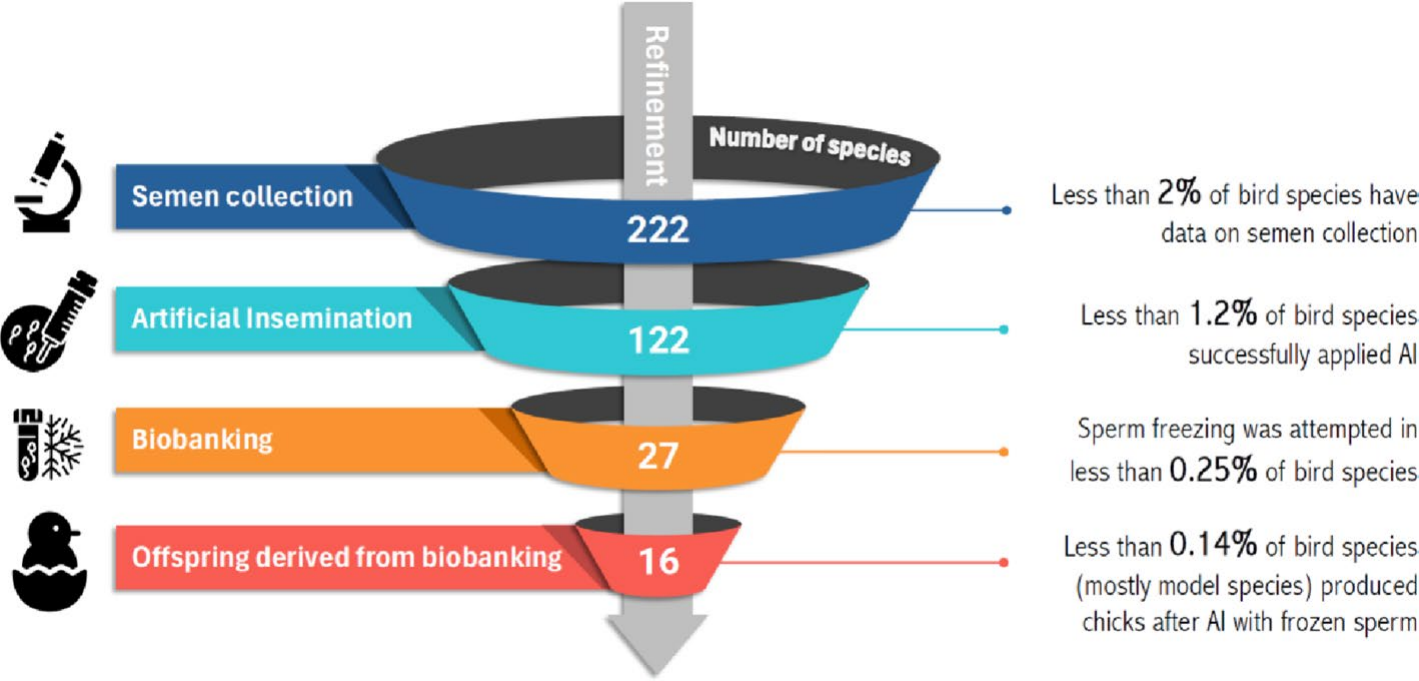
Funnel diagram depicting the number of wild birds covered by semen technologies (STs) according to their refinement, where the top of the funnel represents only semen collection and the bottom the integration of various techniques with artificial insemination using frozen semen with the production of offspring

Another important aspect is the number of species covered by each ST and how many of them are classified as at risk of extinction according to the IUCN (Fig. 2). This assessment emphasized that STs have been conducted in less than 2% of all existing bird species, of which only 59 (0.5%) are listed as threatened to some degree. The breakdown of this data in terms of refinement of the techniques applied makes the figures even more shocking, considering that AI (largely carried out with fresh semen) reached less than 1.2% of avian species, most of which were not endangered (Fig. 3). Similarly, of the few species in which AI was performed using frozen sperm (*n* = 16), only two were endangered (*Tragopan blythii* and *Tragopan caboti*) resulting a single chick.

**Fig. 4:**
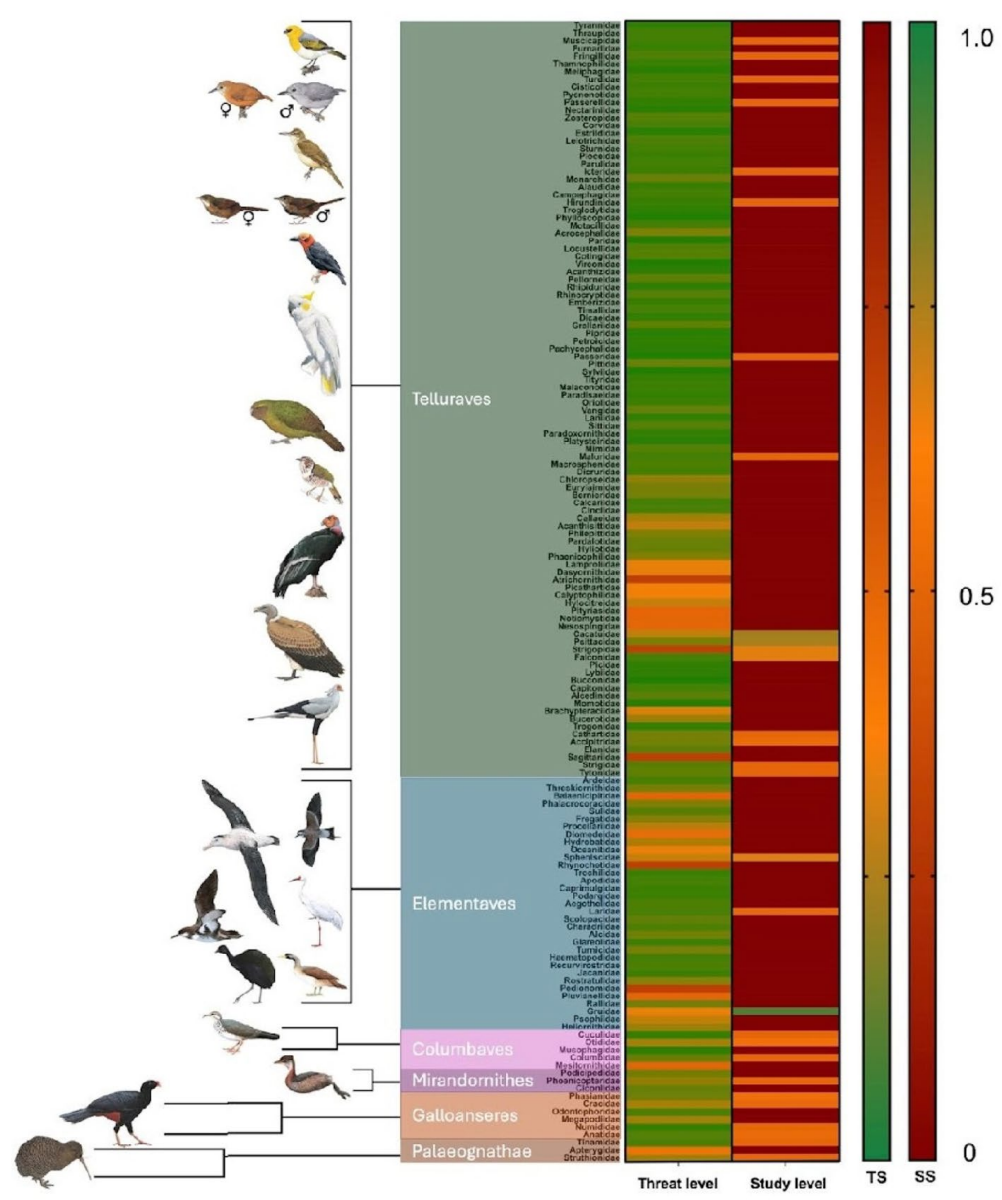
Heat map of the relative threat and study levels (first and second columns, respectively) for each bird family according to the criteria described here in the *Material and Methods* section. Columns TS and SS indicate threat and study scales, respectively. The zero (0) indicates the lowest threat and study levels, whereas one (1) indicates the highest threat and study level

Of the 222 species studied, 133 (59.9%) belong to the Psittacidae, Phasianidae and Cacatuidae families (with 88, 27 and 18 species, respectively). Adding to this estimate the families Gruidae and Accipitridae (with 13 species each), we have 71.6% of all work to date concentrated in just 5 families. In an attempt to better understand the distribution of these studies within different bird families, a heatmap was created showing the total number of threatened species per family alongside the number of species to which STs were applied (Fig. 4). The study scale calculated here for each family was zero for the vast majority of bird families, and for those with some study, the values ranged between 0.35 and 0.70 (excluding the Gruidae, which showed a score of 0.9). This means that, within the few families with some study, the majority of them have a small proportion of species with some type of knowledge or application. This two-dimensional graphical representation of the data also highlighted that, for the most part, STs were aimed at families with low threat levels according to our criteria. For example, despite having threatened species, the Psittacidae (parrots, macaws and conures), Falconidae (falcons) and Accipitridae (hawks and eagles) families have had a large volume of research over the years when compared to families with a higher threat level. In this regard, the Gruidae family (cranes) drew particular attention given the large number of threatened species to which some type of STs has been applied, followed by other families such as Strigopidae, Cacatuidae and Spheniscidae (New Zealand parrots, cockatoos and penguins, respectively). On the other hand, this approach also showed that some families with high conservation priority, such as Atrichornithidae (scrub birds), Sagitaridae (secretary bird), Rhynochetidae (kagu), Pedionomidae (plains-wanderer), Diomedeidae (albatrosses) and Balaenicipitidae (shoebill) have been neglected, leading us to wonder why the STs have not reached these taxa.

**Fig. 5:**
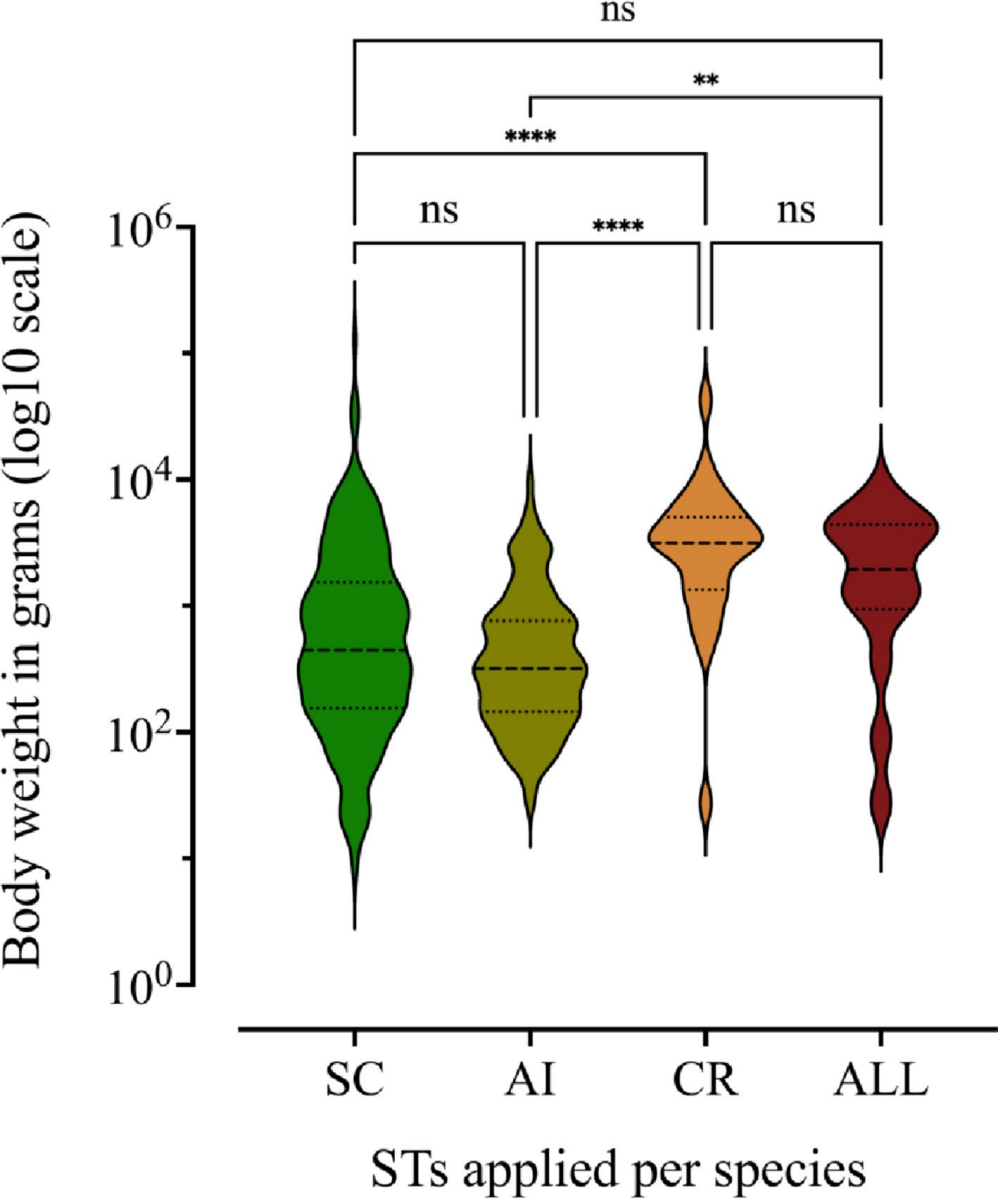
Comparison between the body weights of wild birds used in studies involving semen technologies (semen collection – SC; artificial insemination –AI; sperm cryopreservation – CR; and insemination with cryopreserved sperm – ALL). The data is shown in logarithmic scale, and asterisks indicate significant difference among means (** *P* < 0.01. **** *P* > 0.0001), while ‘ns’ indicates non-significant difference among means (*p* > 0.05)

**Fig. 6:**
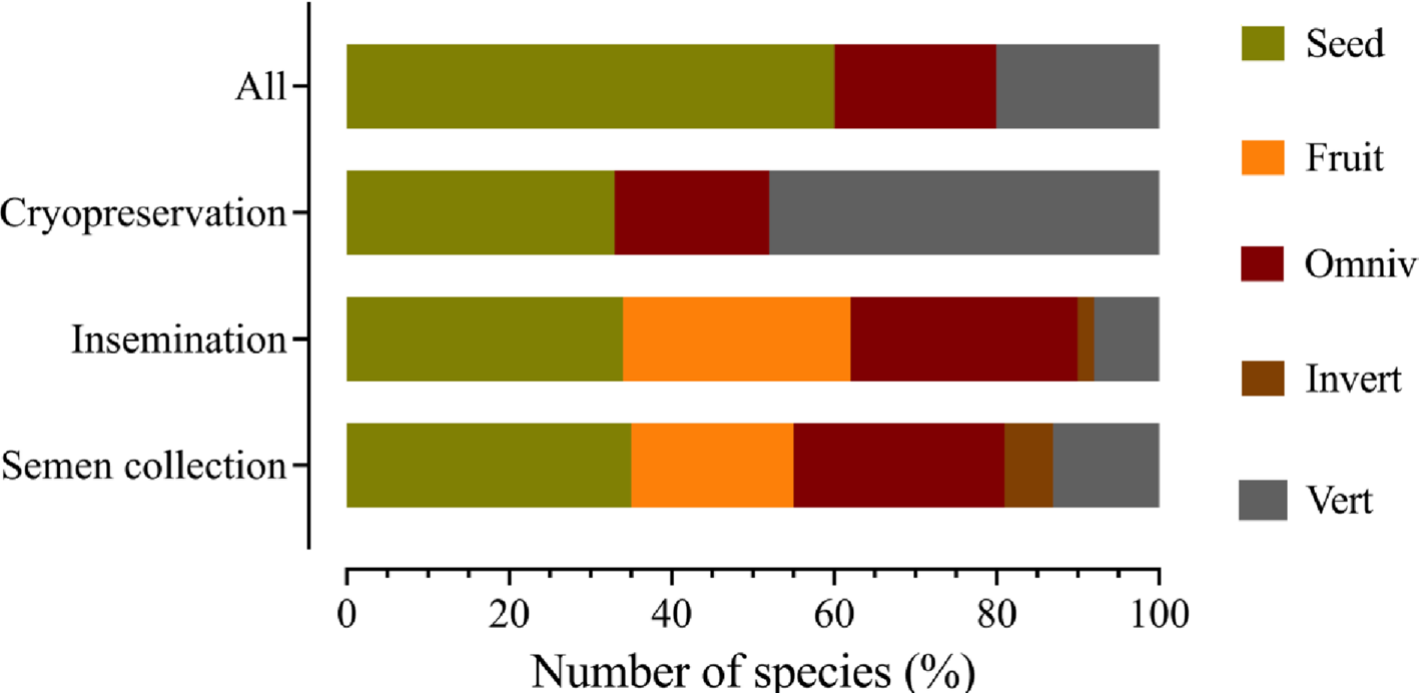
Percentages of bird species with different feeding strategies within each semen technology categories (semen collection, artificial insemination, semen cryopreservation, and artificial insemination with cryopreserved semen - ALL). Seed, Fruit, Ominv, Invet and Vert correspond to species described as seed eaters, frugivorous, omnivorous, invertebrate eaters (mainly insects), and vertebrate eaters (including scavenger birds), respectively

In light of this question, we assessed whether the body weight of birds could be an important factor influencing the application of these technologies (Fig. 5). Our findings demonstrated that semen collection was employed in a wide variety of bird sizes, followed by artificial insemination which, although on average did not show a significant difference, proved to be more restricted in terms of size. Nevertheless, it became clear that studies involving semen cryopreservation and insemination with frozen semen were more prevalent among heavier species. Feeding strategy was another component analyzed, showing research records related to semen collection and artificial insemination for five diet groups defined here (Fig. 6). However, when considering methods more focused on the formation and use of biobanks (i.e., storage and use of frozen semen), we noticed that efforts were exclusively carried out in seed eaters (e.g., Phasianidae, Anatidae and Psittacidae), and vertebrate eaters (Accipitridae, Falconidae and Spheniscidae) and omnivorous birds (Gruidae and Phoenicopteridae), ignoring a wide range of frugivorous, insectivorous birds whereas species exclusively nectivorous as hummingbirds, sunbirds, and honeyeaters were not contemplate in any study, regardless of the STs applied.

## Discussion

Since the first report of semen collection in chickens in the early 20th century (Burrows and Quinn 1935), the use of different semen technologies (STs) has been expanded to various wild birds with the promise of assisting *ex situ* breeding programs in increasing fertility and genetic variability. Our data reveal that from 1960 to 1990 there was less than one published study per year on this topic, and although this average has risen to over 7.2 articles per year in the last two decades, such increase is probably much smaller than the advances in STs in wild mammals over the same period. The possible reasons for this inequality may lie in the shortage of researchers dedicated to avian assisted reproduction worldwide, or the large anatomical and physiological differences in birds that hinder the transposition of existing technologies from mammals (Woods et al. 2022). When scrutinizing the purposes of these studies, we observed that almost half of them were exploratory and related to methods of semen collection or obtaining seminal or sperm characteristics, in other words, without a sequence for the subsequent application of artificial insemination, semen cryopreservation, or both. This information highlights that, despite decades of research, technical refinement of STs in birds has been asymmetrical, with a strong bias toward the acquisition of basic knowledge rather than toward the integration of methods for more concrete conservation results. For example, only 16% of the compiled studies evaluated AI with frozen semen, which indicates very modest progress regarding the formation and, especially, the use of germplasm banks after such a long period of time. This limitation is likely due in large part to the difficulties inherent in freezing avian sperm, which even in poultry remains challenging to this date [Thélie et al. 2019], but also raises the need for other alternatives for the genetic preservation of populations at risk. In addition, there have been very few studies in which free-living birds have been collected or inseminated (even those in basic science), a fact that exposes a strong dependence between research and maintenance of species in captivity. This forces us to rethink how reproductive studies should be developed in the near future, perhaps adapting them more to field conditions, since it is very unlikely that viable populations of most endangered species (or model species for them) can be maintained *ex situ* for experimental purposes.

Unfortunately, it was also found here that only 4.1% of the world’s threatened bird species were addressed in any way by the STs (i.e., 0.5% of the total species known to date). This extremely low number of species benefiting from these techniques can probably be explained by the lack of financial resources and professionals with expertise in the area, the absence of a captive population for testing, or difficulties in accessing individuals in the wild. In parallel, our results reveal that research with STs, and consequently conservation-related funding, has often been directed toward bird families with much lower threat levels than others, because are larger, more charismatic or have greater monetary value, such as parrots, raptors, waterfowl, and pheasants (Psittacidae, Accipitridae, Falconidae, Anatidae, and Phasianidae families, respectively). It is important to note that these families also have threatened species, but proportionally there are taxa that have a much larger number of threatened species or a set of species with worse conservation status in relation to the total number of species, as in the case of families in which their few or only representative is threatened (e.g., scrub birds, kagu, secretary bird, plains wanderer, shoebill, among others species native to islands or highly fragmented habitats). Likewise, our survey found no evidence of ST work involving albatrosses (Diomedeidae) and petrels (Oceanitidae), families that also received high threat scores according to our criteria. It is clear that the challenges in maintaining some of these species in captivity or studying them in the wild ultimately limit not only the development but also the application of STs. Notwithstanding, we have also identified inspiring examples of the use of STs in the conservation of highly threatened families (Fig. 4) such as Gruidae (cranes), Strigopidae (New Zealand parrots), and Cacatuidade (cockatoos).

The fact that research on STs in parrots and birds of prey accounted for almost 60% of the studies surveyed, even though less than 28% of their species are threatened, is not surprising as these taxa have a huge global market because they are seen as pets, service animals or partners in hunting sport (Panter et al. 2023). Thus, high market demand has led to captive breeding and, consequently, the application of STs improving at a faster rate than in other bird groups. As a result of the reproductive success of these commercially bred species, it has become easier to apply these technologies for the conservation of their threatened counterparts (Blanco et al. 2009; Lierz et al. 2013). However, passerines contradict this model: despite being among the most traded taxa worldwide, only 12.5% of their 6,659 species are held in captivity, and fewer than 24% are successfully bred (Wahle et al., 2024). This pattern aligns with our findings (Fig. 4), which show that only 14 passerine species (0.2%) have been studied in detail, with most research focusing on sperm morphometry or other topics not directly related to conservation and breeding. Unfortunately, in this group, STs have had a very limited dissemination, perhaps due to issues associated with semen collection via conventional massage techniques that require prolonged handling. In general, passerines are prone to death from stress caused by handling and the idea or fear of criticism that eventual death of threatened birds occurred due their handling can inhibit the improvement or use of STs in these birds. However, these issues can be significantly minimized when the semen collection is performed by trained staff (Donoghue et al. 2010). Losses of valuable birds can be completely ruled out by using model species or birds imprinted to ejaculate on mannequins or humans (Bailey and Lierz, 2017; Girndt et al. 2017; Łukaszewicz et al. 2015; Lierz et al. 2013a; Samour, 2002). Yet there are species that have no captive congeners to be used as breeding models, making it essential to obtain wild birds in order to start an *ex situ* conservation programme. For instance, in a recent effort to develop captive breeding methods for the critically endangered Alagoas antwren (*Myrmotherula snowi*), whose current populations is estimated at 6 free-living adults, individuals of Plain antivireo (*Dysithamnus mentalis*) and White-flanked antwren (*Myrmotherula* axillaris) were captured in the Amazon and Atlantic rain forest (Vilela et al. 2024). One strategy that has proven to be quite interesting for the establishment of founder populations in birds is the collection of fertile eggs, as this reduces the stress of individuals being kept in captivity and also creates opportunities for the use of STs with imprinted birds. (Anderson, 1990; Boyce et al. 2005; Forbes, 1990). Furthermore, these fertile eggs can also represent a priceless source of cells such as the primordial germ cells (PGCs) which, although still requiring methodological adjustments for their widespread use in conservation, can be propagated in vitro, frozen and later transferred into recipient birds (Tae et al. 2008; Whyte et al. 2015; Chen et al. 2019, 2023, 2025; Gessara et al. 2021; Ballantyne et al. 2021; Jung et al. 2023; Hu et al. 2024; Doddamani et al. 2025).

A valuable debate was previously raised by Pritchard et al. (2012) and Feliciano et al. (2023) on the basis for choosing species to participate in captive breeding programmes, and these authors concluded that taxa eligibility for *ex situ* conservation is not typically defined by the level of threat but by other aspects such as body size, public appeal, diet, ease of management and previous presence in captivity. Given the aforementioned interface between the maintenance of *ex situ* populations and the development of STs, our findings seem to agree with this assertion since the refinement of STs also appeared to be influenced by the body size and feeding strategy of different avian species. Highly specialized or very complex diets cause many zoos and breeders to avoid acquiring individuals of certain species due to the labor and costs inherent in their maintenance. Nutritional deficiencies can negatively impact the health of captive individuals, compromising their efficiency of both natural and artificial reproduction by unbalancing hormones, inhibiting courtship and copulation behaviors, reducing semen quality, etc. (Peters et al. 2004). Another obstacle faced by many captive breeding programmes refers to the occurrence of sexual monomorphism in some avian species. Although this issue may appear minor at first glance, it is more common than often assumed and may compromise conservation efforts, particularly when sexing is based solely on external morphology or when molecular methods have flaws. In practice, we have observed several cases in which individuals of the same sex were mistakenly paired, a problem that becomes even more critical in socially monogamous species, in which pair compatibility is closely linked to reproductive performance. In this context, Frediani et al. (2019) demonstrated that pairing status can significantly affect the success of semen collection in different bird species. Specifically, the probability of obtaining semen was highest in paired males with fertile clutches, compared with unpaired males or males paired without clutches, including males paired with other males. These findings indicate that breeding condition and pair status are important factors influencing collection efficiency and should be taken into account when applying semen technologies to wild birds. We hope that improvements in molecular sexing techniques will reduce these limitations by enabling less invasive and more precise sex determination, thereby facilitating pair formation and reproductive management in the many avian species that exhibit sexual monomorphism (Turcu et al. 2023).

In view of the various threats faced by different bird species around the world, striking a balance between in situ and *ex situ* measures is imperative for the success of conservation programmes. For many taxa, their only chance of long-term survival depends heavily on the efforts and investments in captive conservation plans to increase populations and their respective genetic variabilities through reproduction. In this context, STs, together with breeding management and new technologies (e.g., biobanking, cell culture, germ cell transplantation, cell reprogramming, among others) can greatly assist institutions in achieving this goal more quickly and efficiently. However, the current list of threatened birds is too long to be covered by both *ex-situ* programmes and reproductive technologies. For this reason, prioritization criteria must be well defined in order to use financial and human resources wisely so that the largest number of species can benefit from these tools. We hope to contribute with this data to the discussion of which directions to take so that we can help global avian biodiversity.

## Supplementary Information

Below is the link to the electronic supplementary material.

Supplementary Material 1

Supplementary Material 2

## Data Availability

The authors declare that they have no known compering financial interest or personal relationships that could have appeared to influence the work reported in this paper. The findings and conclusions contained within are those of the authors and do not necessary reflect positions or policies of the Gates Foundations neither the UK Government.
