# Supplementary material for: Are we truly helping those in need: A comprehensive assessment of semen technologies in wild bird species
Source: Biodivers Conserv. 2026 Jun 29;35(8):199. doi: 10.1007/s10531-026-03397-7 (PMC13315471; doi:10.1007/s10531-026-03397-7)

Supplementary file: Are we truly helping those in need: A comprehensive assessment of semen technologies in wild bird species

Marcel Henrique Blank^1*^, Julia Roismann^2#^; Matheus Moraes Azevedo^2#^; Ricardo Jose Garcia Pereira^2^

^1^The Roslin Institute and Royal (Dick) School of Veterinary Studies, University of Edinburgh, Roslin, UK

^2^Department of Animal Reproduction, School of Veterinary Medicine and Animal Science, University of São Paulo, Pirassununga, Brazil

^*^Corresponding author: [mblank@ed.ac.uk](mailto:mblank@ed.ac.uk)

^#^These authors contributed to the manuscript equally

**Supplementary Fig. 1. Visual diagram of the workflow used to construct and analyze the dataset of semen semen technologies (STs) in birds. Literature-derived dataset of semen technologies in wild birds.** Relevant studies were identified through systematic searches, screened according to predefined inclusion criteria, and used to extract information on semen collection, semen analysis, semen storage, cryopreservation, and artificial insemination. The dataset was subsequently combined with taxonomic, ecological, and conservation variables to assess potential biases in research effort and application of semen technologies among bird families.


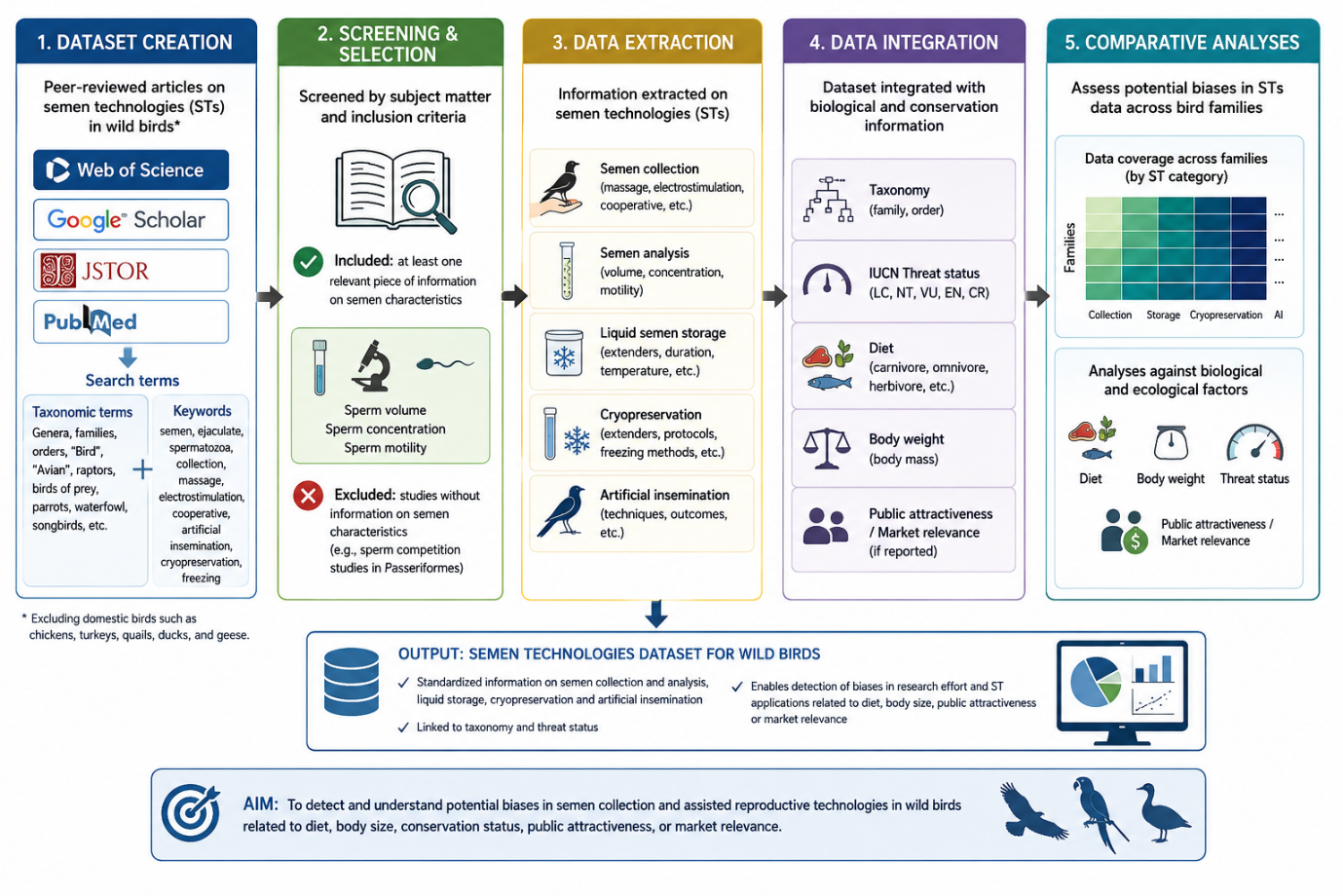

Supplement: Supplementary file 2 — Supplementary Material 2 [file 10531_2026_3397_MOESM2_ESM.docx]
